# Cancer Biology and Prevention in Diabetes

**DOI:** 10.3390/cells9061380

**Published:** 2020-06-02

**Authors:** Swayam Prakash Srivastava, Julie E. Goodwin

**Affiliations:** 1 Department of Pediatrics, Yale University School of Medicine, Yale University, New Haven, CT 06520-8064, USA; 2 Vascular Biology and Therapeutics Program, Yale University School of Medicine, New Haven, CT 06520-8066, USA

**Keywords:** metformin, insulin, thiazolidinediones, incretins, dipeptidyl peptidase 4, sodium-glucose cotransporter 2, AMPK activators, antineoplastic therapy and diabetes, epithelial-to-mesenchymal transition, endothelial-to-mesenchymal transition, catechol-*o*-methyl-transferase, endothelial-cell glucocorticoid receptor, multiomics, PPPM, type I diabetes mellitus and cancer and type II diabetes mellitus and cancer

## Abstract

The available evidence suggests a complex relationship between diabetes and cancer. Epidemiological data suggest a positive correlation, however, in certain types of cancer, a more complex picture emerges, such as in some site-specific cancers being specific to type I diabetes but not to type II diabetes. Reports share common and differential mechanisms which affect the relationship between diabetes and cancer. We discuss the use of antidiabetic drugs in a wide range of cancer therapy and cancer therapeutics in the development of hyperglycemia, especially antineoplastic drugs which often induce hyperglycemia by targeting insulin/IGF-1 signaling. Similarly, dipeptidyl peptidase 4 (DPP-4), a well-known target in type II diabetes mellitus, has differential effects on cancer types. Past studies suggest a protective role of DPP-4 inhibitors, but recent studies show that DPP-4 inhibition induces cancer metastasis. Moreover, molecular pathological mechanisms of cancer in diabetes are currently largely unclear. The cancer-causing mechanisms in diabetes have been shown to be complex, including excessive ROS-formation, destruction of essential biomolecules, chronic inflammation, and impaired healing phenomena, collectively leading to carcinogenesis in diabetic conditions. Diabetes-associated epithelial-to-mesenchymal transition (EMT) and endothelial-to-mesenchymal transition (EndMT) contribute to cancer-associated fibroblast (CAF) formation in tumors, allowing the epithelium and endothelium to enable tumor cell extravasation. In this review, we discuss the risk of cancer associated with anti-diabetic therapies, including DPP-4 inhibitors and SGLT2 inhibitors, and the role of catechol-o-methyltransferase (COMT), AMPK, and cell-specific glucocorticoid receptors in cancer biology. We explore possible mechanistic links between diabetes and cancer biology and discuss new therapeutic approaches.

## 1. Introduction

Diabetes mellitus (DM) is characterized by disruption in glucose homeostasis and defects in insulin action on many target tissues including liver, muscle, pancreas, and adipose [1,2,3]. Diabetes is a common metabolic abnormality and is classified as two types: type I is pathologically based on the deficiency in insulin secretion by pancreatic β-islet cells and type II is characterized by insulin-resistance which renders target cells unable to adequately respond to insulin and thus unable to use blood glucose for energy [1,2,3]. To compensate, the pancreas makes increasingly more insulin, resulting in insulin resistance syndrome which includes obesity, high blood pressure, high cholesterol, and eventually type 2 diabetes [2,3]. From a survey of the International Diabetes Federation, there were 366 million people with diabetes in 2011, and the total number is expected to rise to 552 million by 2030. Type 1 diabetes accounts for 5%–10% of the total cases of diabetes and type II diabetes accounts for 90%–95% [4]. DM-on a global scale-is a major threat to public health, healthcare systems and the economy, due to the series of pathologies triggered in a long-term manner after DM manifestation [5]. Specifically, modifiable risk factors should receive particular attention in the context of the currently observed DM epidemic which is predicted to expand to over 600 million diabetes-diseased people by the year 2045 [5]. The innovative approach of predictive diagnostics, incorporating targeted prevention and treatment tailored to the individual with suboptimal health (before clinical onset of disease manifestations), as the medicine of the future is the most prominent option to reverse currently persisting disastrous trends in diabetes care [5].

Prolonged diabetes causes multi-organ dysfunction including nephropathy, retinopathy, neuropathy, atherosclerosis, heart disease and cardiovascular dysfunction [3,6,7]. Epidemiological evidence supports the fact that diabetes and cancer are linked by revealing increased risks of some cancers in several diabetic populations after adjusting for other confounding factors, such as obesity and dyslipidemia [8,9]. Also, epidemiological studies suggest that patients with diabetes who develop cancer have a worse prognosis after treatment with chemotherapy or surgery and have a higher mortality rate than patients without diabetes [9]. Recent data suggest possible mechanistic links between diabetes mellitus and certain types of cancer [8,9]. Indeed, diabetes mellitus and cancer share many risk factors, including aging, hyperlipidemia, obesity, and sex [8]. Both type I and type II diabetes mellitus have been associated with an increased risk of cancer progression [8]. Advanced approaches of differential plasma proteome, detection of circulating RNAs and gene expression studies in circulating leukocytes have been discussed for development of potent diagnostic and therapeutic targets for cancer in diabetes. There is high potential for the application of a gene therapy approach in diabetes care that can be used for gene repair by gene replacement therapies [10].

Cancer is one of the most pressing public health problems [11], but the association of cancer development with diabetes mellitus has been overlooked by scientists. The factors that contribute to this phenomenon are largely due to lack of proper epidemiological data on clinical practices and the lack of special guidelines for cancer screening in diabetic subjects [11]. However, recent discoveries about the possible reduced incidence of cancer development in patients treated with metformin, a well-known anti-diabetic drug, have forced both endocrinologists and oncologists to reconsider the mechanistic links between diabetes mellitus and cancer [12,13,14]. 

Cancers share similar phenotypes with diabetes such as higher insulin and Insulin-like growth factor 1 (IGF-1) or leptin/adiponectin secretion and immune abnormalities. Cancer cells increase the use of glucose to sustain high proliferation [15]. Maximized nutrient demand and disturbed metabolic shift in cancer cells induce metabolic adaptations in neighboring non-cancer cells [15]. Finally, these metabolic shifts compromise the organ function of adipose, liver, and muscle, leading to cachexia, a metabolic syndrome featuring typical diabetic features and responsible for 20% of cancer deaths [16,17]. However, the molecular mechanisms of cancer in diabetes are currently not clear. Disturbed metabolic homeostasis causes increased generation of reactive oxygen species and oxidative damage to nuclear and mitochondrial DNA frequently observed in diabetic patients. Long term accumulation of damaged DNA or DNA mutations is well-known to trigger cancer. Impaired DNA repair is known as a highly energy consuming process and leads to mitochondrial dysfunction. Mitochondrial dysfunction is implicated in mechanisms of diabetes-provoked cancer. There is a growing evidence that diabetes mellitus predisposes individuals to almost all cancer types with some particular preferences [18].

## 2. Prevalence of Cancer in Diabetes

Two-large-cohort studies in subjects, consisting of 30,000 patients each, suggest an association between type I diabetes mellitus and cancer [19,20]. In the first study, Zendehdel et al. found that the risk of cancer, especially of the stomach, cervix, and endometrium, was twenty percent higher in type I diabetic subjects [19]. The second study, by Swerdlow et al., showed that the prevalence of ovarian cancer was doubled in type I diabetic subjects below thirty-years-of-age, and that type I diabetes had the highest risk for those patients diagnosed between the ages of ten and nineteen years [20]. Another study revealed that the incidence of pancreatic cancer is higher in type I diabetic subjects [21]. Type I diabetes is an auto-immune disease that is often associated with an increased risk in cancer progression [22]. However, there is a need for further scientific research to explore the link between type I diabetes mellitus and cancer progression [22]. 

Most clinical studies have examined the risk of cancer in patients with type II diabetes [23]. For instance, a potent mitogen, insulin-like-growth-factor (IGF)-1, demonstrates higher levels in type II diabetic subjects and may contribute to cancer progression [24]. Studies also suggest an association between type II diabetes and cancer in many organs, such as the endometrium, breast, stomach, colorectum, pancreas, liver and blood [8]. Also, risks for gallbladder and biliary cancer are higher in type II diabetic subjects [25]. In contrast, the prevalence of prostate cancer is decreased in type II diabetic subjects [26]. It is known in animal models that hyperinsulinemia accelerates breast cancer progression [23] and IGF-1 induces hepatic cancer in adenocarcinoma models [24]. Insulin resistance in type II diabetics is critically-linked with an excess accumulation of diacylglycerol in cells that leads to activation of protein-kinase C, a well-known influencer in cancer cells [27,28,29,30]. Type II diabetes mellitus is often associated with dyslipidemia and obesity, which further enhance the risks of cancer progression [31]. Dysglycemia and hyperinsulinemia are possible mechanisms through which diabetes mellitus promotes tumor growth and tumor metastasis [8]. In addition, several other factors are linked to diabetes mellitus and cancer metabolism, including oncogenes and tumor-suppressor-genes, glutamine metabolism, inflammation, dyslipidemia, and obesity [8]. 

Type II diabetes is associated with most site-specific cancers linked to obesity and positively correlates strongly with endometrium and kidney and weakly with bladder, prostate and stomach cancer [17,32]. Importantly, lung cancers are inversely related with obesity and diabetes [17]. Type I diabetes is also linked with site-specific cancers and associated with endometrial and stomach cancer more strongly than type II diabetes [17]. The association with type I diabetes and type II diabetes was found to have a similar magnitude for cancers of the pancreas and thyroid, and leukemia [17]. Type I diabetes is positively associated with gastrointestinal, blood, thyroid and bladder cancer whereas melanoma, kidney, prostate, and ovarian cancer are inversely related. Importantly, breast cancer doesn’t associate with type I diabetes [17]. Evidence suggests a positive association of obesity with cancer; importantly, prostate cancer associates positively and inversely to type II diabetes, whereas lung cancers inversely associate with obesity, but not with type II diabetes [17,32]. Therefore, a generalized approach to study the association between cancer risk and diabetes or obesity may be faulty since the most prevalent cancers are not linked (lung), or inversely linked (prostate), with diabetes [17,32]. Moreover, some neoplastic treatments, such as glucocorticoids, may induce diabetes, and should be explored in depth. 

## 3. Antidiabetic Drugs in Cancer

### 3.1. Insulin and Insulin Analogs

Hyperinsulinemia and hyperglycemia are central components regarding the link between diabetes and cancer [33]. The insulin receptor is a tyrosine kinase which exists in two isoforms: IR-A and IR-B. IR-B is expressed primarily in insulin-sensitive tissues and signals its metabolic effects through activation of the phosphoinositide 3-kinase pathway [33]. IR-A is expressed in fetal tissue and cancer cells, and signals cell survival and proliferation through the Ras-mitogen-activated protein kinase (MAPK) pathways [34]. Both receptors signal through activation of insulin receptor substrate (IRS) family proteins, including IRS-1. IRS-1 overexpression has also been shown to have oncogenic effects through promoting cell proliferation, inhibiting basal and oxidative stress-induced autophagy, and ultimately decreasing cell death in NIH/3T3 fibroblasts [35]. Long term human insulin and insulin analogue use contributes to diabetes-associated cancers through activation of insulin receptors [36]. Studies in cultured cells indicate that short-acting analogs offer biological effects that are similar to those of insulin [37]. However, long-acting analogs, such as glargine and detemir, have been found to have a slow binding to receptors for insulin but a higher binding to IGF-1R, that cause activation of the ERK pathway, and an increased mitogenic effect in respect to insulin [37]. Retrospective epidemiological clinical studies have suggested that long-acting analogs use may increase the risk for cancer [38]. Insulin-glargine is a well-known long-acting insulin analog that is given for basal insulinization with a lower risk of hypoglycemia [36]. However, clinical data have suggested an interesting association between insulin–glargine and risk of cancer progression. Hemkens et al. found that, considering the connections between insulin doses and cancer progression [36], the cancer incidence with insulin-glargine use was higher than that when using endogenous insulin [39], especially in the case of prostate and breast cancer [39,40,41]. Tseng et al. observed that insulin users who also had chronic obstructive pulmonary disease had the highest risk of lung cancer when compared to patients without insulin use and without chronic obstructive pulmonary disease, (adjusted hazard ratio: 1.891, 95% confidence interval: 1.767–2.024) [38]. Moreover, another study recruiting postmenopausal women suggested that diabetes increased the risk of lung cancer (hazard ratio: 1.27, 95% confidence interval: 1.02–1.59), which was more remarkable among patients treated with insulin (hazard ratio: 1.71, 95% confidence interval: 1.15–2.53) [42]. Wu et al. concluded that insulin was associated with an increased risk of lung cancer in patients with diabetes mellitus (odds ratio: 1.23, 95% confidence interval: 1.10–1.35) [43].

However, But et al., investigated the use of certain insulins and risk for cancer, addressing the limitations and biases involved in previous studies [44]. No trend with cumulative treatment time for insulin glargine relative to human insulin was observed in risk for any of the ten studied cancer types [44]. Of the 136 associations analyzed in the main analysis, only a few increased and decreased risks were found: among women, a higher risk was observed for colorectal (RR 1.54, 95% CI 1.06, 2.25) and endometrial cancer (RR 1.78, 95% CI 1.07, 2.94) for ≤0.5 years of treatment and for malignant melanoma for 2–3 years (RR 1.92, 95% CI 1.02, 3.61) and 4–5 years (RR 3.55, 95% CI 1.68, 7.47]). Among men, a lower risk was observed for pancreatic cancer for 2–3 years (RR 0.34, 95% CI 0.17, 0.66) and for liver cancer for 3–4 years (RR 0.36, 95% CI 0.14, 0.94) and >6 years (RR 0.22, 95% CI 0.05, 0.92) [44]. These data suggest that there is no evidence of consistent differences in the risk for ten cancers following insulin glargine or insulin detemir treatment compared with human insulin, at follow-up exceeding five years [44]. To date, the US Food and Drug Administration has not finalized that insulin-glargine treatments increase the risk of cancer advancement, though the safety review is still ongoing [45]. New long-acting insulin, known as degludec, has been developed, and it will be important to analyze the effects on cancer risk, if any [46].

### 3.2. Sulfonylureas

Sulfonylureas (SU) are the class of antidiabetic drugs that are most widely used in the management of diabetes mellitus [47]. SU are secretagogues for insulin and are reported to be associated with a higher risk of cancer development. Currie et al. demonstrated that type II diabetic patients who were treated with SU monotherapy displayed an increased incidence of cancer development, similar to that of insulin-treated patients [47]. However, the higher incidence of cancer in SU-treated subjects was improved by co-administration of metformin [47]. A population-based cohort study demonstrated that patients treated with SU showed an increased rate of cancer-associated mortality, which was identical to that found in insulin-treated patients, as compared to the mortality rate in patients treated with metformin alone [48]. Specific types of SU are associated with diverse rates of cancer incidence. A retrospective observational cohort analysis by Monami et al. found that cancers in diabetic subjects dosed with glibenclamide displayed remarkably higher mortality rates and an increased risk of cancer as compared to that of diabetic subjects dosed with gliclazide [49,50].

A literature search of diabetic subjects and cancer revealed six studies, of which three were retrospective and three were prospective [49,50,51,52,53,54]. Five of the six studies focused on all-cancer incidence [49,51,52,53,54] and one reported both all-cancer and site-specific cancer incidence [50]. Two of the six studies had all-cancer mortality as the primary outcome and the other four had cancer incidence as the primary outcome [49,51]. Three of the six studies analyzed dose response relationships for each SU, and one of the six studies investigated treatment duration response differences for each SU [50,52,53,54]. In these six studies, data on cancer risk were found for gliclazide, glimepiride, glibenclamide, and tolbutamide use, while no data were observed for the other SU (chlorpropramide and glipizide) [55].

### 3.3. Metformin and Cancer

Metformin, which is a well-known medication for the management of type II diabetes, seems to suppress the risk of cancer [56]. Evans et al. reported a lower incidence of cancer progression in a diabetic population treated with metformin [12]. Bowker et al. executed a five-year follow-up study of 12,309 diabetic patients and found that metformin-dosed patients had decreased cancer-related mortality as compared to patients treated with insulin or SU [48]. Metformin treatment has been shown to be associated with extended lifespan and a reduction in the incidence of cancer [57]. However, in a collaborative meta-analysis of randomized clinical trials, Stevens et al. did not find significant beneficial effects of metformin on cancer outcomes [58]. 

Metformin diminishes ATP levels that result in an increased ratio of AMP-to-ATP, leading to activation of the liver-kinase-B1 (LKB1)-AMP-activated-protein kinase (AMPK) signaling pathway [59,60,61]. Metformin inhibits hepatic glucose production in an LKB1- and AMPK-independent manner [62,63]. Metformin potentially inhibits cancer cell growth through targeting diverse biological pathways [60,61,63,64]. The anti-cancer properties of metformin have been analyzed in several experimental models [61,65,66]. Metformin inhibits tumor growth in high-fat diet-fed mice [67]. These studies suggest metformin can be a safe candidate drug for preventing tumor growth in the diabetic population. However, the beneficial effects of metformin on cancer cells have not always been supported by data from retrospective clinical studies using pathological endpoints [68,69]. Thus, long-term, randomized prospective studies are needed to confirm the potential benefit of metformin.

In addition to metformin’s well-established antidiabetic properties, there has been considerable interest in its antitumor properties. However, this interest arose from a short observational study which suggested that the use of metformin was linked with a 23% lower risk of any cancer [12]. After that, a large number of observational studies have been analyzed “corroborating” a possible lower risk of cancer with metformin [70]. This apparent convergence of evidence from both observational and laboratory studies has led to a call for large randomized clinical trials (RCTs) of metformin in cancer prevention and treatment [69,71,72]. Suissa et al., analyzed that thirteen observational studies suffered from immortal-time bias, nine studies had not reviewed time-window bias, while other studies did not consider inherent time-lagging issues when comparing the first-line treatment metformin with second- or third-line treatments [73]. These studies are subject to time-related biases that are preventable with proper study design and data analysis which then lead to unreal, extraordinarily significant results, with reductions in cancer risk with metformin ranging from 20% to 94% [73]. However, a careful assessment of the observational studies conducted to date points to some important time-related biases that systematically exaggerated the reported antitumor effects of metformin [73]. Time-related biases, such as immortal time bias, time window bias, and time lag bias, have been previously described in studies of diabetes treatment [73]. These biases result from not properly classifying exposure during the follow up of a cohort study or from measuring exposure over uneven time intervals in case-control studies, which can generate misleading risk reductions [74]. 

The study conducted by Mamtani et al. [75] was an observational study that joins a growing series of observational studies reporting no effect of metformin use on cancer prevention and treatment [76,77,78,79]. These studies may have other biases, but are generally free from major time-related biases that potentially exaggerate the benefits of metformin. With the study by Mamtani et al. [75], the evidence is now mounting against an association between metformin and cancer, so that a careful reassessment is now warranted before more RCTs of metformin as a treatment for cancer are initiated [74]. 

In this line, Oh et al., conducted a study to investigate the association of metformin therapy with the development of cancer [80]. A total of 66,627 adult subjects with type II diabetes were included in the analysis; 29,974 were metformin users and 36,653 were controls [80]. After multivariable adjustment, the risk for the development of cancer among metformin users was not significantly different from that among controls (HR = 0.96; 95% confidence interval, 0.89–1.03; *p* = 0.250), suggesting a lack of association between metformin therapy and the risk of cancer among patients with diabetes [80]. Feng et al., conducted a meta-analysis of cohort studies to evaluate a potential association of metformin use with prostate cancer risk [81]. Eighteen cohort or nested case-control studies were included with a total of 52,328 cases. In a random-effect pooled analysis, metformin use was not significantly associated with the risk of prostate cancer (RR 0.97, 95% CI 0.80–1.16, *p* = 0.711) [81].

### 3.4. Thiazolidinediones, Peroxisome Proliferator-Activated Receptor-γ and Cancer

Thiazolidinediones (TZD) are another drug class used to treat type II diabetes [82]. TZD works as an agonist of the nuclear receptor peroxisome proliferator activated receptor-γ (PPAR-γ) and enhances insulin sensitivity [82]. PPAR-γ mediates cell cycle arrest and has tumor suppressor activity in liposarcoma, lung, and prostate cancers; and inhibits colonic polyp formation in adenomatous polyposis coli (APC) min/+ mice. Available studies show that TZD suppresses the growth of cancer cells in vivo and in vitro [83,84,85,86]. In humans, seventeen trials (three case-control studies and fourteen cohort studies) excluded a cancer risk with TZD treatment [87]. However, a mild risk of bladder cancer was found, especially in those treated with pioglitazone [87]. There was no correlation observed with pancreatic, lung, breast, prostate, or kidney cancers.

To assess the influence of TZDs, Govindarajan et al., conducted a retrospective analysis of a database from 10 Veterans Affairs medical centers. Of 87,678 subjects, 1137 had colorectal cancer, 3246 had prostate cancer, and 1371 had lung cancer. Govindarajan et al., observed a 33% reduction in lung cancer incidence among TZD treatment in diabetic patients compared with non-users (relative risk, 0.67; 95% CI, 0.51 to 0.87), however, the risk reduction for colorectal and prostate cancers post- TZD treatment did not reach statistical significance [88]. An epidemiological study showed that diabetes mellitus comorbidity adversely affects lung cancer outcomes [89] however, there was no association nor increased risk of lung cancer in type II diabetic patients found [32,90]. 

A total of 606,583 type II diabetic patients without a history of cancer were identified from the Taiwan National Health Insurance [91]. A significantly lower risk of liver cancer incidence was found with any use of rosiglitazone (OR: 0.73, 95% CI: 0.65–0.81) or pioglitazone (OR: 0.83, 95% CI: 0.72–0.95), suggesting that pioglitazone and rosiglitazone reduce the incidence of hepatic cancer in type II diabetic subjects [91]. For colorectal cancer, rosiglitazone, but not pioglitazone, was associated with a significantly reduced risk (OR: 0.86; 95% CI: 0.76–0.96). Furthermore, Chang et al. found that TZDs were not associated with lung and bladder cancer incidence, however a higher risk for bladder cancer with pioglitazone use ≥3 years could not be excluded (OR: 1.56; 95% CI: 0.51–4.74) [91]. A meta-analysis using randomized clinical trials to assess the safety studies of rosiglitazone in diabetic patients showed no link with cancer incidence. However, most of the participants enrolled had undergone less than a year of TZD treatment [92]. A longer observation time is likely required to evaluate the safety of TZD [93].

### 3.5. Incretin Drugs and DPP4 Inhibitors in Cancer

Incretins belong to the group of gastrointestinal hormones that cause a postprandial increase in insulin levels secreted by the β-cells, even before blood glucose levels are elevated [94]. In 2011, Elashoff et al. found that pancreatic cancer was more commonly found among patients who were receiving doses of a glucagon-like peptide-1 (GLP-1)-based drug molecule. This finding raises caution about the long-term actions of incretins in the development of pancreatic cancer [93]. In 2013, Butler et al. also found that incretin therapy caused a remarkable secretion of both exocrine and endocrine pancreatic compartments [95]. Exocrine factors increase proliferation and dysplasia, and endocrine factors cause α-cell hyperplasia [95]. The glucose-dependent insulinotropic polypeptide (GIP), and GLP-1, belong to the family of incretins [96]. GIP exerts its effect through a dedicated 7-transmembrane G protein-coupled receptor (GIPR) that is widely distributed in the tissues such as in the pancreas, gastrointestinal tract, vascular endothelium, adipose tissue, and brain [97,98]. On ligand binding, GIPR activates adenylyl cyclase activity, and hence there is an increase in intracellular cyclic adenosine 3′,5′-monophosphate (cAMP). GIP-GIPR signaling also has proliferative and anti-apoptotic effects, via the activation of mitogen-activated protein kinase, through the PI-3K-dependent activation of Akt-PKB [99]. The poor expression of GIPR in adipose tissue is linked to insulin resistance and obesity [100]. Besides its association with metabolic diseases, the GIP-GIPR axis is also gaining interest because of its inappropriate expression and activation in some human endocrine tumors, as the GIP-GIPR axis may be involved in tumor development and therefore, offers a potential target for a new therapeutic approach [101]. Researchers assessed GIPR expression in a broad spectrum of gastrointestinal and bronchial tumors and showed that GIPR expression was higher in neuroendocrine tumors [96,102]. The cause of GIPR gene overexpression in pituitary adenoma has been investigated using several approaches, however, clear molecular evidence is still lacking [101]. The enhanced expression of GIPR on the cancer cell’s surface, and its weaker expression in surrounding healthy tissues underscore this receptors great potential as a molecular target for both imaging and radiation therapy in neuroendocrine tumors [102,103]. This was confirmed by two distinct proof-of-principle studies using xenograft neuroendocrine tumor mouse models and two different GIP-based radioactive tracers [101,104,105].

GIPR expression varies in adrenal and pituitary tumors, and neuroendocrine tumors, but - regardless of the tumor’s type and origin, cAMP signaling is activated in all GIPR-positive tumors and not in GIPR-negative ones [101]. Further studies are required to select pathways activated by the GIP in these neoplasms, and to establish whether and how they can affect tumor behavior [101]. The contribution of the GIP/GIPR axis can be further investigated in endocrine tumors, such as in functional neuroendocrine tumors, that synthesize, store and secrete peptide hormones in a cAMP-dependent manner, as findings could have potential diagnostic and therapeutic implications [101]. The presence of GIPR-positive endocrine tumors should be considered when determinimg incretin mimetics for the treatment of type II diabetic patients [101].

GLP1 is degraded in vivo by the enzyme dipeptidyl peptidase-4 (DPP4), which is a 110-kDa cell surface glycoprotein, also well-known as CD26. GLP1 has multiple functions in tumor behavior, depending on the tumor type and the tumor microenvironment [95]. DPP4 is a key drug target for the therapy of type II diabetes and diabetic complications [106,107,108].

The tumorigenic role of DPP4 is variable in different tumors [109]. In tumors, such as astrocytoma, gastrointestinal stromal tumors, and some lymphomas, higher expression of DPP4 is linked with tumor aggressiveness [110,111]. In contrast, the absence or loss of DPP4 expression is observed in the advanced stage of certain malignancies, including melanomas, endometrial carcinoma, and lung squamous cell carcinoma [112,113]. Overexpression of DPP4 in urothelial carcinoma correlates with tumor cell growth, proliferation, and enhanced cell migration and invasion [114]. Suppression of DPP4 attenuates aggressiveness and promotes apoptosis in urothelial carcinoma cells [114].

DPP4 inhibition causes higher levels of both endogenous GLP1 and GLP2 [115]. DPP4 plays an important role in cancer progression and metastasis [116,117]. However, the long-term use of DPP4 inhibitors for the treatment of type II diabetes has been debated and one study suggests that patients with type II diabetes treated with a DPP4 inhibitor do not have a higher risk of developing cancers than patients treated with a placebo or other drugs [118]. An analysis based on the US Food and Drug Administration adverse event reporting system database reported increased risks of pancreatic cancer with sitagliptin treatment. The rate for pancreatic cancer was 2.7 times higher with sitagliptin than with other therapies (*p* = 0.008) [93]. Two large-scale randomized controlled trials (RCTs), Saxagliptin Assessment of Vascular Outcomes Recorded in Patients with Diabetes Mellitus-Thrombolysis in Myocardial Infarction 53 (SAVOR-TIMI 53) and Trial Evaluating Cardiovascular Outcomes with Sitagliptin (TECOS), were conducted to assess the cardiovascular safety of saxagliptin and sitagliptin, respectively [119,120]. The results of the two trials indicated that there was no significant increase in the risk of pancreatic cancer. Interestingly, a protective effect of saxagliptin against colon cancer was found in the SAVOR-TIMI 53 trial (hazard ratio = 0.51, 95% CI = 0.27–0.92, *p* = 0.026) [121].

There have been many trails to assess the efficacy of DPP4 inhibition in diabetic subjects. A meta-analysis by Monami et al. evaluated the effect of DPP4 inhibition on developing pancreatic cancer [122]. A recent study confirmed that DPP4 inhibition did not increase tumor occurrences but may promote the metastasis of multiple cancer cell lines [123]. Through enzymatic reactions, DPP4 regulates the activity of biopeptides by proteolytically cleaving many peptides, cytokines, and chemokines [124]. C-X-C Motif Chemokine Ligand 12 (CXCL12) also known as stromal cell-derived factor 1 (SDF1), is a known substrate of DPP4 [125]. CXCL12 binds to the receptors C-X-C Motif Chemokine Receptor 4 (CXCR4) and C-X-C Motif Chemokine Receptor 4 (CXCR7) and thus regulates tumor growth and tumor metastasis [126]. In breast cancer, the CXCL12/CXCR4 axis plays a crucial role in directing the metastasis of CXCR4-positive cancer cells to organs that express high CXCL12 levels, such as the lungs, bone marrow, and lymph nodes [127]. Therefore, higher CXCL12 levels in response to DPP4 inhibitor treatment can be relevant to the metastasis of CXCR4-positive cancers [128]. Incidentally, elevated DPP4 levels are found in less invasive epithelial ovarian carcinoma [129].

**Figure 1:**
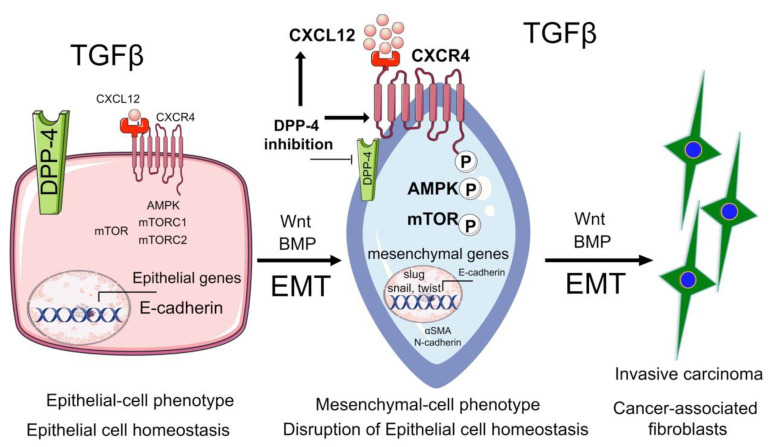
Role of DPP4 in cancer biology. In normal diabetic epithelial cells, DPP4 is highly expressed and is associated with lower levels of the CXCL12/CXCR4/mTOR/EMT axis. Inhibition of DPP4 induces TGF-β- independent activation of CXCL12/CXCR4/mTOR/EMT axis, promotes the formation of cancer-associated fibroblasts and accelerates cancer metastasis.

However, an anti-CD26 monoclonal antibody treatment showed anti-tumor properties in vitro and in vivo in lymphoma and renal carcinoma [130]. In CD-1 mice, DPP4 inhibition did not induce dysplasia in the colon and showed no tumor-promoting activities [131]. In 2013, Femia et al. reported that long-term treatment with the DPP4 inhibitor (sitagliptin) reduced features of colon cancer and reactive oxygen species in rats, and that the protective nature of DPP4 inhibition against colon cancer could be utilized in chemoprevention clinical trials [132]. One trial showed that DPP4 inhibition by another DPP4 inhibitor (saxagliptin) was not associated with an increased incidence of cancers [133]. Aoe et al. found that a higher level of CD26 expression is linked to a better response to chemotherapy [134]. In contrast, Yang et al., describe that inhibition of DPP4 accelerates breast cancer metastasis through induction of the CXCL12/CXCR4, which activates mammalian-target-of-rapamycin (mTOR) to induce EMT [135]. Figure 1 depicts that DPP4 plays a significant role in cancer biology and that inhibition of DPP4 promotes cancer metastasis via induction of the CXCL12/CXCR4/mTOR/EMT axis [135]. The epithelial cells from diabetic mice have shown activated levels of Wnt, BMP and TGFβ signaling when compared to non-diabetic epithelial cells [135,136,137]. Activation in Wnt, BMP, and TGFβ signaling are known to stimulate EMT processes [135]. However, its direct association with CXCL12 or with DPP4 is not known and is a matter of ongoing research.

mTOR, a key molecule in the PI3K/Akt pathway, is linked to glucose-sensing and autophagy and is involved in malignant transformation [138]. mTOR occurs in 2 complexes: mTORC1 (carrying mTOR, Raptor) and mTORC2 (carrying mTOR, Rictor). mTORC1 is sensitive to rapamycin, while mTORC2 is rapamycin-insensitive [139]. A recent finding implicated mTORC1 and mTORC2 as key regulators of EMT, and knockdown of mTORC1 and mTORC2 induced mesenchymal-to-epithelial transition, while inhibition of mTOR signaling suppressed cancer cell migration and invasion [140]. Moreover, crosstalk regulation between the mTOR pathway and the CXCL12/CXCR4 axis suggests that mTORC1 silencing is sufficient to decrease CXCR4-mediated cancer cell migration, and inhibition of mTORC1 by rapamycin decreases primary tumor growth and CXCR4-mediated lymph node metastasis [141].

### 3.6. Sodium-Glucose Cotransporter 2 Inhibitors in Cancer

Sodium/glucose cotransporter 2 (SGLT2) is a protein that is encoded by the *SLC5A2* (solute-carrier-family 5 (sodium/glucose cotransporter) gene in humans which is the major cotransporter involved in glucose reabsorption in the kidney tubules [142]. SGLT2 inhibitors are a class of glucose-lowering drugs useful for treating type II diabetes mellitus [143]. SGLT2 inhibitors decrease plasma glucose levels by selectively inhibiting renal glucose reabsorption and increasing urinary glucose excretion [144,145]. Also, SGLT2 inhibitors offer other benefits such as weight loss and reduction in blood pressure [146]. In clinical practice, SGLT2 inhibitors are prescribed in combination with metformin and/or other agents as second-line therapy (combination therapy) if an individual fails to attain glycemic control with one or more other antidiabetic agents [147].

Tang et al., studied a meta-analysis of all available head-to-head randomized controlled trials to assess whether SGLT2 inhibitors affect cancer risk in type II diabetic patients [148]. The same group further performed a network meta-analysis to evaluate the comparative effects of SGLT2 inhibitors on cancer risk [148]. Current evidence from short-term trials did not indicate an increased risk of cancer in the type II diabetic population using SGLT2 inhibitors. Given the short-term trial duration, however, future long-term prospective studies and post-marketing surveillance studies are required [148].

A recent pooled analysis of twenty-one trials suggested that the increased risk of bladder and breast cancers observed in those treated with a specific SGLT-2 inhibitor (Dapagliflozin), might be the absence of detailed diagnosis prior to randomization rather than a causal relationship [149]. An elevated risk of bladder or breast cancer has not been reported for other SGLT2 inhibitors in humans [150], though they may induce tumors in rodents [151,152]. Given the conflicting results about possible links with rare cancers, individual trials are not enough to delineate the cancer risk associated with the use of SGLT2 inhibitors. Apart from the clinical data, SGLT2 inhibitors have shown anti-cancer effects and reduced tumor growth in renal cell carcinogenesis in vitro and in vivo [153].

A few meta-analyses indicate that SGLT2 inhibitors are not linked with a significantly increased risk of cancer [148,154]. One pooled analysis of 21 phase 2b/3 clinical trials demonstrated that the incidence of malignancies was similar between a dapagliflozin group and the comparator groups [149]. In addition, the overall risks of bladder, breast and renal cancers was not increased by canagliflozin in a pooled analysis of eight phase 3 clinical trials [150]. Moreover, preclinical studies did not support increased hyperplasia in the urinary bladder mucosa, urogenital tract or kidney in SGLT2-deficient mice compared with control mice [155]. Furthermore, in one study, a non-significant risk increase among individuals using SGLT2 inhibitors with a lower CI limit of 0.96 (OR 1.17 [95% CI 0.96, 1.41]) was observed [148]. However, these cannot completely rule out the possibility of an increased cancer risk [148]. These findings require further analysis in large trials such as CANVAS (canagliflozin; NCT01032629) and DECLARE-TIMI58 (dapagliflozin; NCT01730534), as well as in long-term observational studies [148].

Interestingly, evidence from meta-analyses displayed that canagliflozin was significantly associated with a decreased risk of gastrointestinal cancer [148]. SGLT1 and SGLT2 have been found to be overexpressed in many cancers such as pancreatic and prostate adenocarcinomas [156,157]. SGLTs, especially SGLT1, plays a key role in cancer cell survival [157]. Canagliflozin is not only a potent SGLT2 inhibitor but also has potent SGLT1 inhibitory activity [156]. SGLT1 is found mainly in the gastrointestinal tract, but also in the kidneys and heart, while SGLT2 is mainly expressed in the kidneys and less so in the gastrointestinal tract [158]. Therefore, canagliflozin might protect against gastrointestinal cancer by suppressing the expression of both SGLTs. Further prospective studies are needed to determine the potential effects of SGLT2 inhibitors on the risk of gastrointestinal cancer.

A higher risk of bladder and breast cancer is always a safety issue linked with SGLT2 inhibitors [148]. A pairwise meta-analysis demonstrated that SGLT2 inhibitors (empagliflozin) was significantly associated with bladder cancer [148]. The risk of bladder cancer was identified from the EMPA-REG OUTCOME Trial (empagliflozin: six incidences of bladder cancer, two incidences of bladder transitional cell carcinoma and one incidence of bladder cancer recurrent; placebo: zero incidences) [159]. Increased risk of bladder cancer was found in the patients taking empagliflozin as compared to placebo [159]. However, one of meta-analysis studies showed significantly increased risk of bladder cancer with dapagliflozin or canagliflozin [148]. The mechanisms underlying the elevated risk of bladder cancer linked with SGLT2 inhibitors remain unclear. Diabetes and obesity are indeed risk factors for bladder cancer, and increased rates of glycosuria and urinary tract infections related to SGLT2 inhibitor use may be responsible for the observed increased risk [150].

In brief, the available RCTs do not demonstrate a significant link between SGLT2 inhibitors and an increased risk of overall cancer. There is some evidence available suggesting that SGLT2 inhibitors (empagliflozin) might increase the risk of bladder cancer, whereas canagliflozin might offer a protective effect against gastrointestinal cancer [148]. However, given the short-term design of the RCTs included in the analysis, the long-term effects of SGLT2 inhibitors on cancer remain uncertain. Future long-term prospective studies are warranted.

**Figure 2:**
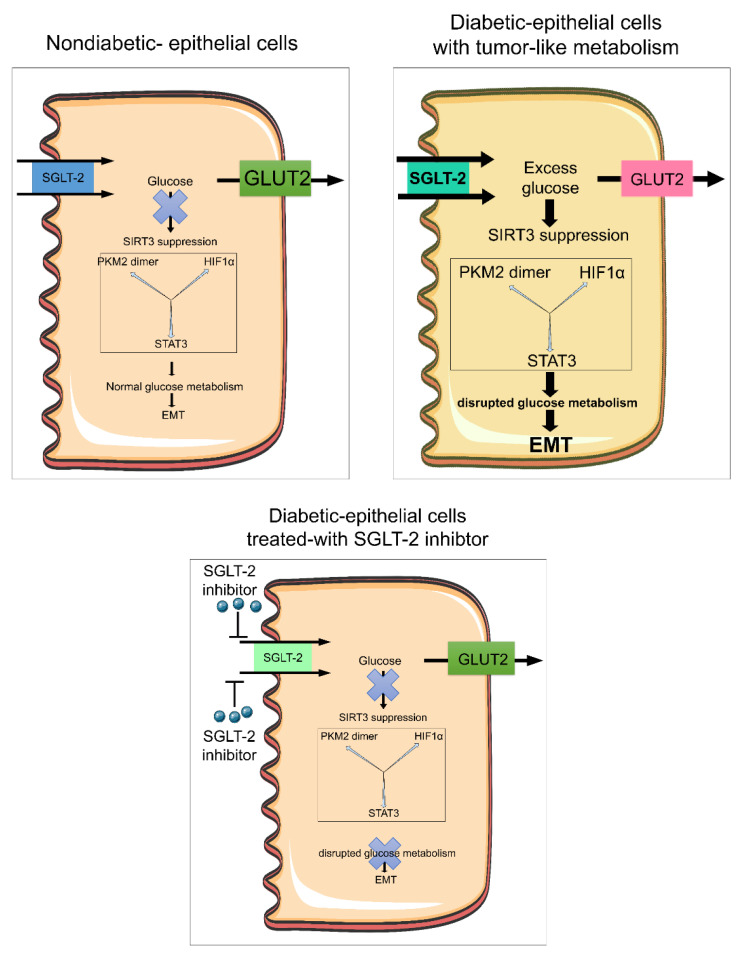
Pathological significance of urinary glucose in the disruption of glucose metabolism. SGLT-2 is highly expressed in diabetic epithelial cells. The function of SGLT-2 is to absorb urinary glucose which can then be reabsorbed into the blood, however, in severe diabetes, this excess glucose accumulates in the cytosol, activates SIRT3-deficiency-associated induction of augmented glycolysis and suppresses fatty acid metabolism. Accumulation of PKM2, HIF1α, and STAT3 phosphorylation play a key role in the disruption of central metabolism, a phenotype that is similar to the Warburg effect in tumor cells. This disruption in central metabolism leads to epithelial cell injury and promotes EMT processes. SGLT-2 inhibition abolishes these effects.

Figure 2 depicts a hypothetical scheme showing the critical role of SGLT2 in aberrant glucose metabolism and associated EMT in diabetic kidneys. In this figure we describe diabetic tubular epithelial cells (TECs) that express higher levels of SGLT-2 on the luminal side and GLUT2 on the basolateral side [160,161]. In diabetic epithelial cells, glucose is absorbed from the urine into the cell through SGLT-2 and this absorbed glucose is transported efficiently out of tubular cells by GLUT2 [160,161]. In diabetes mellitus, higher expression of SGLT-2 increases the absorbing capacity of glucose from urine into TECs, as compared to the ability of GLUT2 to transport the glucose out of TECs [160]. In this diabetic condition, glucose accumulates in the cells, which results in the suppression of SIRT3 [161]. The subcellular distribution of SIRT3 is disturbed which results in less SIRT3 in the mitochondria and cytosol [161]. Moreover, a suppressed level of SIRT3 in the cytosol leads to defective glucose metabolism, by inducing PKM2 dimer formation, HIFα accumulation and STAT3 phosphorylation [160,161,162]. Such mechanisms are similar to the Warburg effect observed in tumor metabolism. Treatment with an SGLT-2 inhibitor (empagliflozin) significantly suppressed this defective metabolism and EMT processes [160].

In addition, SIRT3 deficiency leads to deacetylation of enzymes of fatty acid oxidation and causes suppression in fatty acid oxidation; some studies have demonstrated that SIRT3 deficiency is involved in mitochondrial DNA fragmentation which is released through mitochondria into the cytosol and causes inflammation [163,164]. This process also supports our hypothesis since defective fatty acid metabolism contributes to the induction of EMT and EndMT processes [161,165].

## 4. Cancer Therapies and Diabetes

Cancer therapies (radiation, biological agents, and chemical) target the immune system, pyrimidine analogues target replicative immortality, cisplatin targets apoptosis, and mTOR inhibitors alter cell signaling and affect tumor metabolism [17]. Some of these therapies may lead to transient or permanent diabetes. Two major therapies, targeting cancer growth have been associated with hyperglycemia; mTOR inhibitors (Everolimus and Temsirolimus) and tyrosine kinase inhibitors (Nilotinib and Pazopanib) [17]. Everolimus causes hyperglycemia in 12% of renal cell carcinoma, in 5% of pancreatic or gastrointestinal cancers and in 4% of breast cancer subjects [17,166]. Nilotinib causes hyperglycemia in 5% of treated patients with chronic myeloid leukemia [167]. Inhibitors targeting PI3K/AKT signaling also induce hyperglycemia [168]. Up to 8.4% of subjects treated with a PI3K inhibitor (BKM120) displayed hyperglycemia. Targeting IR or IGF-1R directly resulted in a higher percentage of patients developing hyperglycemia. Patients treated with PI3K/AKT/mTOR pathway inhibitors developed hyperglycemia of grade 3–4 (6.7% vs. 0% of controls) [168]. High dose glucocorticoids are often used as adjuvants in cancer therapy, but can cause hyperglycemia and insulin resistance [169]. Androgen-deprivation therapy is utilized to treat prostate cancer. Moreover, testosterone suppression is linked to insulin resistance [170]. Only 12.5% of patients taking androgen deprivation therapy developed insulin resistance [171]. As the frequency of prostate cancer is lower in diabetic men, demonstration of hyperglycemia while on androgen deprivation therapy might be more significant. Pyrimidine analogues, (5-Fluorouracil) which inhibit RNA synthesis and cause DNA damage, are used to treat colorectal and pancreatic cancers [172]. 5-Fluorouracil induced hyperglycemia occurs in 26.1% of CRC patients and 13.2% went on to develope diabetes [169]. Cisplatin enhances cancer cell apoptosis and is used to treat a variety of cancers. Cisplatin caused transient diabetes in 5% of patients in two cohorts of head and neck cancer patients [173,174]. Radiotherapy was found to associated with diabetes [175]. However, the molecular mechanisms that support this outcome remain unclear.

## 5. Biology of EMT in Cancer

EMT is characterized by a series of processes through which epithelial cells lose their epithelial features and acquire mesenchymal cell properties [176,177,178,179]. Epithelial cells are associated tightly with neighboring cells, which inhibit their potential to dissociate from the epithelial layer. In contrast, mesenchymal cells do not form a layer of cells or intercellular adhesion complexes [180]. Mesenchymal cells are elongated in shape, have polar ends and show focal adhesions, allowing for increased migratory capacity and invasiveness [180]. EMT is essentially involved in several developmental processes including mesoderm and neural-tube formation. Epithelial cells express high levels of E-cadherin, whereas mesenchymal cells express *N*-cadherin, fibronectin, and vimentin. Thus, EMT leads to profound morphological and phenotypic cellular changes. In adults, the primary function of fibroblasts, which are prototypical mesenchymal cells, is to maintain structural integrity by secreting extracellular matrix (ECM). Fibroblast-specific protein 1 (FSP-1), alpha-smooth muscle actin (αSMA), fibronectin, and collagen I are markers which characterize the mesenchymal products generated by EMT [180,181,182]. Inflammatory injury results in the recruitment of a diverse array of cells that can trigger EMT through the release of growth factors, such as transforming-growth factor-beta (TGFβ), platelet-derived growth factor, epidermal growth factor, and fibroblast growth factor-2 [181,182,183].

EMT is classified into three biologically distinct types [181]. Type I EMT is associated with embryo formation and organ development and is a process organized to generate diverse cell types that share a common mesenchymal phenotype. Type I EMT produces mesenchymal cells that can subsequently go through a mesenchymal-to-epithelial-transition (MET) process to generate secondary epithelia [181]. Type II EMT is linked to wound healing, tissue regeneration, and organ fibrosis [136]. Type II EMT normally generates fibroblasts to repair tissues following trauma and inflammatory injury. However, in contrast to type I EMT, type II EMT is linked to inflammation. Tissue fibrosis is an abnormal form of wound healing, due to prolonged inflammation [161,181]. Diabetes-associated increased rates of type II EMT are involved in organ fibrosis, including in the kidney [160,162]. Diabetes-associated type II EMT induces mesenchymal features in neighboring cells including perivascular endothelial cells [160]. Type III EMT occurs in neoplastic cells that have to go through genetic and epigenetic transformations in genes that favor clonal outgrowth and development of localized tumors. Type III EMT influences oncogenes and tumor suppressor genes. Carcinoma cells undergoing type III EMT invade, metastasize, and generate the final manifestations of cancer progression [181]. Type III EMT contributes to the accumulation of cancer-associated fibroblasts.

Type III EMT is associated with increased cancer cell motility, metastasis, and chemotherapeutic resistance [181] and offers resistance to oncogene-associated premature senescence. Twist1, Twist2, and ZEB1 protect human cells and mouse embryonic fibroblasts from senescence. TGFβ promotes tumor invasion and evasion of immune surveillance at advanced stages. TGFβ also acts on activated Ras-expressing mammary epithelial cells, favoring EMT [184]. Evidence suggests that epithelial cells undergoing EMT develop stem cell-like features, hence generating cancer stem cells. ZEB1 can confer stem cell-like properties, thus constructing a correlation between EMT and stemness. Therefore, the induction of EMT not only favors carcinoma cells to enter the bloodstream, but also endows them with properties of stemness that increase tumorigenic and proliferative potential [185].

Not all cells can go through complete EMT processes, which include losing their cell-cell adhesion and gaining migration characteristics that can affect disease phenotype; instead, most cells undergo partial EMT, a transition state in which they carry epithelial traits such as cell-cell adhesion or apicobasal polarity, and gain migratory features. Cells in this hybrid epithelial/mesenchymal (E/M) phenotype are endowed with special features such as collective cell migration [186,187,188]. Two mathematical models have been demonstrated to explain the emergence of the hybrid E/M phenotype, and it is highly likely that different cell lines adopt different hybrid-state(s), as evidenced by experiments in MCF10A, HMLE and H1975 cell lines [186,187]. Although this hybrid E/M state has been demonstrated as ‘metastable’ or transient, recent experiments in H1975 cells suggest that this state can be stably maintained by cells [188].

## 6. Biology of EndMT in Cancer

Vascular endothelial cells can also originate fibroblasts by going through a phenotypic transition, referred to as endothelial-to-mesenchymal transition (EndMT) [165,180,189,190,191]. EndMT is characterized by the loss of endothelial markers, including cluster of differentiation 31 (CD31) and vascular endothelial-cadherin (VE-cadherin), and gain of mesenchymal proteins including αSMA [165,180,189,191,192]. Diabetes-associated EndMT contributes to cardiac fibrogenesis [192,193], pulmonary fibrosis [194,195], idiopathic hypertension [196,197], and corneal fibrosis [198,199,200]. Many signaling pathways that govern EMT also regulate EndMT in the embryonic heart, during the development of cardiac fibrosis [201,202], pulmonary fibrosis [202,203], in liver fibrogenesis [202,204], in renal fibrosis, and in diabetic kidney disease [108,180,189,191,200,205,206].

Endothelial cells demonstrate a set of biomarkers including VE-cadherin, CD31, tyrosine kinase with immunoglobulin-like EGF-like domains 1 (TIE1), TEK receptor kinase (TIE2), von Willebrand factor (vWF), and cytokeratins [180]. During the process of EndMT, biochemical changes lead to decreased expression of endothelial markers and gain-of-mesenchymal markers such as FSP-1, αSMA, smooth muscle 22-alpha (SM22α), *N*-cadherin, fibronectin, vimentin, types I and III collagen, nestin, cluster of differentiation, 73 (CD73), matrix metalloproteinase-2 (MMP-2), and matrix metalloproteinase-9 (MMP-9) [180,207,208]. miR-21, TGFβ, Wnt/β-catenin, and DPP4-β1 integrin are positive regulators of EndMT whereas the FGFR1-miR-let-7 axis and crosstalk between miR-29 and miR-let-7s are negative regulators of EndMT, which affect several diseases including renal fibrosis, atherosclerosis, and diabetes [107,108,136,209,210,211,212,213].

Solid tumors are a complex of cancer cells, endothelial cells, inflammatory cells, and fibroblasts [214]. Endothelial cells contribute to the pool of cancer-associated fibroblasts (CAFs) by EndMT [214]. EndMT accelerates CAF formation in tumors, affects the endothelium to enable tumor cell extravasation, and generates pericyte-like cells within tumors [214,215]. Pericytes play crucial roles in blood vessel maturation, blood barrier maintenance and vessel integrity and function by interacting with endothelial cells [215].

## 7. Biology of Catechol-*o*-Methyltransferase in Cancer

Catechol-o-methyltransferase (COMT) is an enzyme responsible for the catechol metabolism of catecholamines and catechol-estrogens. Estradiol is catalyzed to hydroxyestradiol, one of the catechol-estrogens, through cytochrome P450 [216]. Hydroxyestradiol is the substrate for COMT enzyme, and COMT transmethylates hydroxyestradiol to 2-methoxyestradiol (2ME) [216]. Regarding the physiological significance of 2ME, deficiency in 2ME and COMT leads to a preeclampsia-like phenotype in mice [216] and shows anti-inflammatory properties in vivo and in vitro [217]. The human COMT gene shows functional SNPs, which decrease protein stability and reduce enzymatic activity (COMT^158Val-Met^) [218]. COMT^158Val-Met^ has been associated with many psychiatric diseases [218], and studies have also suggested that COMT^158Val-Met^ participates in obesity and diabetes mellitus [219,220,221,222]. COMT rs4680 high-activity G-allele was found to associate with a lower HgbA1c level and protection from type II diabetes [223]. Metabolic defects are also characteristic of preeclampsia [216,222]. Such risk of metabolic defects in preeclamptic women can be interpreted by the idea that vascular damage during pregnancy causes endothelial cell injury that leads to metabolic defects associated with cardiovascular dysfunction [224,225]. Kanasaki and Srivastava et al., reported that deficiency in COMT leads to disruption of glucose homeostasis in mice and that such metabolic defects could be partially explained by a deficiency in 2-ME [222]. COMT deficiency is a shared molecular mechanism between preeclampsia, metabolic syndrome and type II diabetes [222]. COMT deficiency or gene polymorphism in COMT is important only in cancer in women. The etiology of breast cancer in young women has displayed differences in terms of inheritance, carcinogenesis, and prognosis as compared to that of their older counterparts, possibly indicating distinct biological origins of the disease [226]. Moreover, besides germ-line mutations in the breast cancer susceptibility genes (BRCA1 and BRCA2), little is known about other factors that are implicated in breast carcinogenesis in young women. Most of the known risk factors, including the age at menarche and menopause, age at first full-term pregnancy, and the number of parturitions, are indicators of cumulative estrogen exposure. Researchers have analyzed polymorphisms in genes encoding for enzymes involved in estrogen metabolism, which may predispose to breast cancer [227]. One SNP in the CYP17 gene displays an association between genetic variants and breast cancer risk [228]. Other studies of genetic polymorphisms that involved the biosynthesis or metabolism of endogenous and exogenous carcinogens have demonstrated an association between altered breast cancer risk and tumor progression [229,230].

Estrogen exposure is a crucial risk factor for breast cancer [231]. One critical feature of estrogen is its mitogenic ability in hormone sensitive tissues, such as the uterus and breast. Recently, research has been focused on the cancer-causing abilities of estrogen metabolites. These estrogen metabolites are mediated through activation of estrogen receptor signaling, interactions with other receptors or effector molecules, or binding to DNA [232]. Among all estrogen metabolites, the two catechol-estrogens, 2-hydroxyoestradiol (2-OHE2) and 4-hydroxyoestradiol (4-OHE2), have displayed the most diverse biological effects [232,233,234]. The 4-hydroxyolated form binds to, and activates, estrogen receptor signaling with the same affinity as does estradiol. However, the interaction with the hormone receptor is remarkably reduced for 2-OHE2, which possesses weak hormonal potency [232,234]. Both in vivo and in vitro studies have shown that 4-OHE2 promotes cell proliferation and carcinogenesis [234]. Liehr and Ricci found higher 4-OHE2 levels in human breast cancer tissues [235] and further, both 2- and 4-OHE2 have been reported to experience metabolic oxidation to the highly reactive estrogen-derived semiquinones and quinones [236,237]. These metabolites are possible candidates in carcinogenesis, and interact with DNA, form intermediate adducts and generate superoxide ions in the semiquinones/quinones redox cycling phenomenon [232,237]. These superoxide ions have been reported to damage both DNA and other cellular constituents and such damage is an important event in the etiology of human cancers [238].

One of the crucial inactivation pathways of 2- and 4-OHE2 is by O-methylation [232,236]. These methylated metabolites are more lipophilic, have longer half-lives than OHE2, and have weak binding affinity to the estrogen receptor [232]. Interestingly, 2-methoxyoestradiol (2ME) has been observed to impact carcinogenesis by inhibition of endothelial cell proliferation and migration. This O-methylation of 2- and 4-OHE2 is catalyzed by the enzyme COMT [236]. COMT activity is higher in the liver and kidneys, and it is also expressed at significant levels in the brain, red blood cells, uterine endometrium, and the mammary glands. The COMT gene, located on chromosome 22q11.1–q11.2, has a single nucleotide polymorphism (G→A) in codon 158/108 of the membrane-bound/cytosolic form [239]. The single nucleotide transition causes an amino acid shift, from Val→Met, that determines high- and low-enzyme activity alleles [239]. The COMTMet allele, encoding for the low-activity and heat-labile enzyme, has proved to be 4–5-fold less effective in methylating catechol substrates in vitro [240]. Accumulation of 4-OHE2, due to decreased COMT activity, is hypothesized to confer increased risk for breast cancer suggesting that a polymorphism in COMT is associated with increased risk of breast cancer, however, some studies suggest that a COMT polymorphism is not linked with breast cancer [227,232,237,241].

Activation of AMPK is one of the known treatment options for type II diabetes mellitus [242]. AMPK activation in the liver, skeletal muscle, and adipose tissue stimulates glucose, lipid uptake and metabolism [242]. AMPK is likely one of the targets of COMT protein in association with the pharmacological function of metformin, although metformin treatment shows AMPK-independent effects and AMPK-mediated insulin secretion, is still controversial [222,243]. COMT is one possible target of metformin for its antidiabetic action [222]. In vivo effects of metformin on enhanced insulin secretion were not shown to have a direct effect on the insulin producing β cells [222]. 2-ME induces β-cell survival signaling and induces insulin secretion, by activating PDX-1, and suppressing MST-1 under high-glucose conditions in cultured β-cells [222].

**Figure 3:**
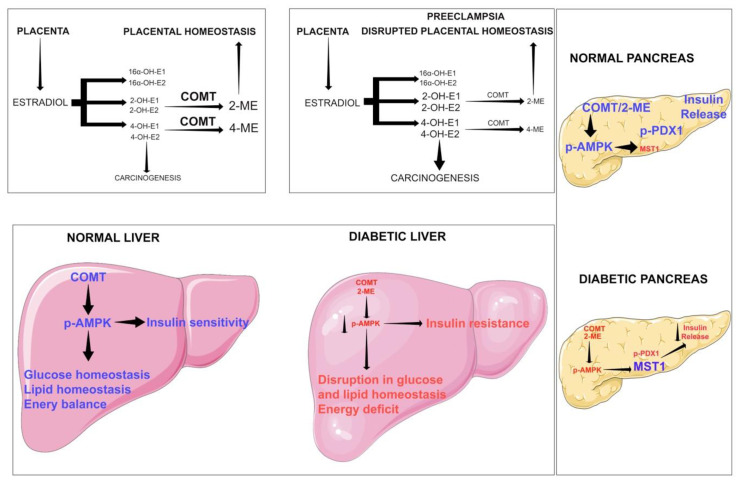
Significance of COMT in regulating metabolic homeostasis. Catechol-*o*-methyltransferase (COMT) is an enzyme that transmethylates hydroxyestradiol into 2-methoxyestradiol (2-ME). 2-ME is protective in maintaining placental homeostasis and metabolic homeostasis through insulin release from pancreatic β-cells. COMT/2-ME phosphorylates liver AMPK which is linked to glucose homeostasis. COMT/2ME is suppressed in the diabetic liver and is linked to a disruption in glucose homeostasis. In pancreatic β-cells, COMT/2ME is an essential protein for PDX1 phosphorylation and is associated with insulin release. In diabetic conditions, low levels of COMT/2ME repress PDX1 phosphorylation and induce MST1. Activated MST1 leads to β-cell death and is responsible for a reduction in insulin release. Cumulatively, COMT is an essential protein that regulates metabolic insults. Deficiency of COMT results in low levels of 2-ME and the accumulation of 4-hydroxyoestradiol (4-OH-E1 and 4-OH-E2). 4-OH-E1 and 4-OH-E2 are believed to possess malignant properties in several organs.

Figure 3 illustrates the protective role of COMT in maintaining placental, liver and pancreatic homeostasis. COMT-product 2-ME maintains placental and metabolic homeostasis by elevating phosphorylation of AMPK in liver and pancreas [222]. COMT/2-ME phosphorylates liver AMPK and improves glucose homeostasis [222]. In pancreatic β-cells, COMT/2ME causes PDX1 phosphorylation which is critical for insulin release [222]. In diabetes, suppressed COMT/2ME levels repress PDX1 phosphorylation and induce serine/threonine protein kinase (MST1) [222]. MST1 phosphorylation causes β-cell death and therefore, reduction in insulin release [222]. Cumulative results suggest that, COMT is an essential protein that regulates metabolic insults [222]. Deficiency of COMT results in low levels of 2-ME and the accumulation of 4-hydroxyoestradiol (4-OH-E1 and 4-OH-E2). In addition, 4-OH-E1 and 4-OH-E2 are believed to possess malignant properties in several organs.

## 8. Biology of AMPK in Cancer

AMP-activated protein kinase (AMPK) maintains cell homeostasis and is a master regulator of metabolism [244]. AMPK is a key molecule in type II diabetes [244]. AMPK has shown tumor suppressor abilities [245,246]. The aim of this review article is to highlight the critical role of AMPK in diabetes and cancer and to analyze its effectiveness in prevention of these diseases. Several experimental studies reported the association between AMPK and metabolic reactions, mainly regulating energy processes. These studies suggest that the effect of AMPK on cell cycle arrest is an important factor for carcinogenesis [244]. Moreover, some clinical studies analyzed cancer prevention targeting AMPK, however, the effective yields in clinical trials have been limited [247,248]. AMPK activation by metformin was shown to improve diabetes and metabolic syndrome and has become a well-established treatment strategy for these disorders. Growing evidence suggests that AMPK is a promising target in the treatment and prevention of cancer cells. Further investigations, including long-term clinical trials with large sample sizes, are needed [244,247,248]. Downregulation of AMPK in tumors involves the insulin/IGF1-regulated protein kinase Akt/PKB, which is hyper-activated in many tumors by gain-of-function mutations in PI3K or loss-of-function mutations in PTEN [247,249]. AMPK suppression was found in human melanoma cells carrying the B-Raf^V600E^ mutation [247,250]. This mutation activates B-Raf, causing activation of the downstream kinases Erk and RSK, which promote phosphorylation of sites in the C-terminal domain of LKB1 that appear to reduce its ability to activate AMPK [247]. Intriguingly, epidemiologic studies provide evidence that prolonged use of AMPK activators protects against cancer development. Thus, patients with type II diabetes taking metformin have a lower incidence of cancer [12].

However, the contribution of AMPK to cancer pathogenesis is not clear since it shows both tumor-suppressing as well as tumor-promoting functions [248,251,252,253]. Apart from the tumor-suppressive abilities, AMPK activation and PGC-1α have been analyzed for their tumor-promoting activity [254,255]. Considering the burden of severe stress in tumor microenvironments, cancer cells must develop a mechanism to overcome stress for their survival. Cancer cells show diverse metabolic responses to the fluctuating microenvironment. During nutritional stress, AMPK maintains energy balance by minimizing energy expense and promoting ATP generation that favors cancer cell survival [242]. The metabolic homeostasis to nutritional stress in cancer cells is primarily caused by the activation of AMPK. Activation of AMPK is linked to an increase in the activities of mitochondrial enzymes and mitochondrial biogenesis in rat skeletal muscle [256,257]. AMPK activation is an important step for regulating mitochondrial biogenesis and cell survival in stress conditions [257]. AMPK controls mitochondrial biogenesis in cancer cells to induce the metabolism of non-glucose carbon sources by controlling p38/PGC-1α [255]. AMPK-p38-PGC-1α regulates energy levels, which are crucial for cancer cell survival in nutrient-deficient conditions [255]. AMPK acts as a metabolic switch, induces glycolysis by activating phosphofructokinase-2, and facilitates mitochondrial metabolism of non-glucose carbon sources, thus maintaining cellular ATP levels [255].

## 9. Biology of Glucocorticoid Receptors in Diabetes and Cancer

Hyperglycemia-associated glucocorticoid receptor (GR) controls the transcriptional regulation of genes that are important for many biological functions such as tumor growth and metastatic progression [258]. GR levels are higher in drug-resistant and metastatic breast cancer cells [259]. GR contributes to tumor cell invasion and lung metastasis in mice [260]. Cancer patients are often treated with glucocorticoids as part of therapy, which may induce hyperglycemia [260]. Both ectopic expression and knockdown of GR show that GR is a strong inducer of EMT [258]. GR suppresses the expression of insulin receptor substrate-1 (IRS-1) by acting as a transcriptional repressor [258]. GR has an antagonistic effect on the expression of IRS-2, suggesting that GR regulates IRS-1 and IRS-2 expression. The GR–IRS-1 axis plays a significant role in regulating the survival and metastasis of breast cancer cells [258]. GR influences cancer cell physiology indirectly through metabolic changes such as impeding glucose and lipid uptake to protect from ER stress, invasion and inflammation [259,261]. Contradictory conclusions about the effect of GR on cancer progression and prognosis in breast cancer have been reported [262,263].

GR is an important contributor to cardiac development, contraction, circadian rhythm and blood pressure management [259]. In diabetes mellitus, steroid treatment is not preferred as several studies have shown that glucocorticoid (GC) treatment contributes to metabolic syndrome [264]. The expression of the ligand-binding GR in skeletal myoblasts is positively linked with metabolic syndrome. Higher levels of GR expression in myoblasts from diabetic subjects suggest higher sensitivity of their skeletal muscle to circulating GC. Excessive GR activation is deleterious to pancreatic β-cells [265]. High-fat-fed β-cell-specific-GR conditional knockout mice show a significant lowering in glucose-stimulated insulin secretion, correlating with abnormal glucose metabolism [266]. Another study demonstrates that hepatocyte cell-specific GR loss suppresses the development of hyperglycemia in streptozotocin-induced diabetes mellitus, due to the aberrant induction of gluconeogenesis, suggesting that liver-specific GR is critical in the development of hyperglycemia and GR antagonists can control hyperglycemia in mice [267]. However, obesity, insulin resistance, and type II diabetes do not require intact GR in adipocytes [268]. The exogenous GR activator dexamethasone promotes metabolic dysfunction, and this effect is less pronounced in mice deficient in GR in adipocyte cells [268]. Activation of GR promotes the whitening of brown fat [268], suggesting that GR plays a role in normal adipose physiology [268].

**Figure 4:**
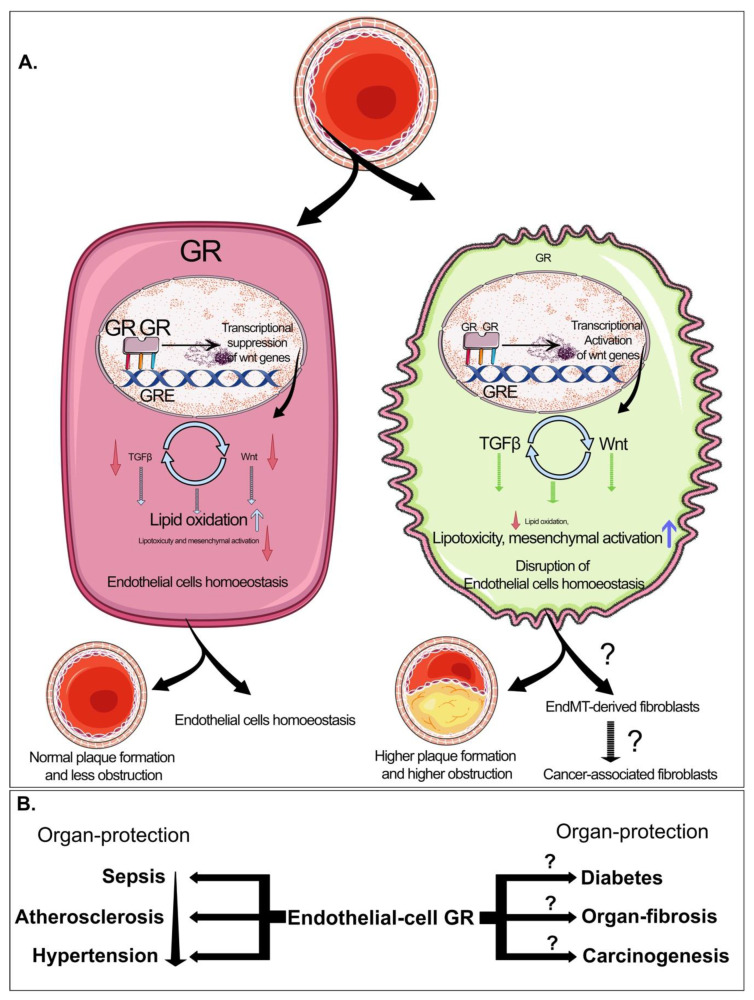
Functional significance of glucocorticoid receptor in endothelial cells. (**A**). In normal endothelial cells, in the presence of GCs, GR binds to GREs and activates the transcription and trans-repression of genes responsible for canonical Wnt signaling. Suppressed Wnt/TGF-β signaling leads to lipid homeostasis and low levels of EndMT, therefore contributing to endothelial cell homeostasis. In diabetic endothelial cells, GR level is suppressed, leading to transcriptional activation of genes in the canonical Wnt signaling pathway. Higher levels of Wnt-dependent TGF-β signaling lead to disruption in lipid homeostasis and higher levels of EndMT, thereby disrupting endothelial cell homeostasis. (**B**). Flowchart showing the role of endothelial cell GR in organ protection.

Physiologically, endogenous GC (corticosterone in rodents and cortisol in humans) perform a role in mitigating local and systemic inflammation. Use of exogenous GC, such as hydrocortisone and dexamethasone (DEX), are widely used to reduce inflammation, though the mechanisms through which they act are not entirely clear [269]. Exogenous GC provides systemic ligand to all cell types expressing GR. Additionally, the side effects of systemic GC are common, rendering them intolerable and often ineffective for vascular inflammatory disorders [270]. In our previous studies, we demonstrated that endothelial cell GR is a negative regulator of vascular inflammation in mouse models of sepsis [271] and atherosclerosis [272]. Mice lacking endothelial cell GR bred onto an *ApoE*^-/–^ background develop more severe atherosclerosis when fed a high-fat diet compared to controls [272]. The increase in the severity of the atherosclerotic lesions cannot be explained by changes in circulating lipids suggesting that circulating cortisol is vasculoprotective. This endothelial cell GR-specific canonical Wnt pathway is independent of NFκB, which is a known classic target for GR, [210,273,274] and therefore signifies the permissive effects of cortisol in endothelial cell health and disease. Figure 4 depicts the role of GR in the regulation of mesenchymal activation and endothelial cell homeostasis. In recent studies, we have demonstrated that in normal endothelial cells, GR binds to GREs and causes suppression in the expression levels of genes responsible for canonical Wnt signaling. In endothelial cells, GR targets canonical Wnt signaling. Suppressed Wnt/TGF-β signaling is known to maintain lipid homeostasis and low levels of EndMT, therefore contributing to endothelial cell homeostasis, suggesting that a normal level of GR in endothelial cells is required for regulation of Wnt and TGFβ signaling. In diabetic endothelial cells, GR is suppressed, leading to transcriptional activation of genes in the canonical Wnt signaling pathway and TGFβ signaling. Higher levels of Wnt-dependent TGF-β signaling lead to disruption in lipid homeostasis and higher levels of EndMT, thereby disrupting endothelial cell homeostasis (Figure 4).

## 10. Perspective and Future Directions

The mechanisms which demonstrate the cancer causing factors in diabetes have been shown to be complex, including excessive ROS-formation, oxidative stress, destruction of several types of essential biomolecules, chronic inflammation, impaired healing phenomena, multiple abnormalities in the levels of DNA, RNA, metabolites, and proteins, collectively leading to carcinogenesis under diabetic conditions.

Innovative approaches, including multiomics, predictive diagnostics, targeted prevention and personalization of medical services, are utilized to systematically analyze carcinogenesis in different biological models [275,276]. Multiomics includes genomics, transcriptomics, proteomics, metabolomics, and radiomics that are more widely used in clinical treatment and in basic research of cancer [275]. The rapid development of several omics technologies has been the driving force to generate multi-omics data. Multi-omics data enhance predictive, preventive, and personalized medicine (PPPM) practices that allow prediction of response with substantially increased accuracy, stratification of particular patients and eventual personalization of medicine [276,277,278]. High performance liquid chromatography, mass spectrometry, and nuclear magnetic resonance technologies are widely used in the discovery of new biomarkers from the cancer proteome and metabolome in diabetes [276]. The PacBio RS and Oxford Nanopore sequencing, with the fundamental feature of single molecule sequencing, is used in genomics. Whereas high throughput RNA seq can cover the entire genome but detect only a few copies of a rare transcript in a cell, RNA sequencing and high resolution sequencing can achieve single base resolution with good accuracy, and are used to analyze the transcriptome analysis in cancer cells in diabetes. These multiomics and molecular network approaches promote to consider cancer from a multiparameter systemic strategy, not from a single parameter model [276]. Moreover, cancer is a complex disease. The integrative multi-omics data provide a holistic view of tumor complexity, and direct the selection of appropriate patients for targeted therapies and evaluation of traditional treatment strategies for improvement in their therapeutic effects. The multiomics technologies have made significant achievements in cancer research which will surely accelerate with the breakthrough of technical limitations [275]. Development of high-throughput, cost-effective multiomics technologies can be extensively used to understand the initiation, progression and efficacy of cancer treatment [275,276].

TGFβ signaling pathway is dysregulated in cancer and has a dual role in different stages of cancer as a suppressor or a promoter [279]. More important, The TGFβ signaling pathway is also another important reason for diabetic complications [280]. In the early stage, TGFβ enforces cell homeostasis by promoting tumor-suppressive effects, including cytostasis, differentiation, and apoptosis [280]. TGFβ exerts tumor suppressive effects by inhibiting inflammation and stroma derived mitogens [280,281]. In the late stage of cancer, mutant TGFβ receptors render cancer cells insensitive to the cytostatic effects of TGFβ [280]. However, cancer cells get benefit from higher tumor release of TGFβ, which promotes cytokine release that in turn promote cell survival. Moreover, TGFβ induces the dependent and independent regulation of EMT, thus promoting the growth of tumors [280]. TGFβ also alters endothelial cell proliferation, migration, and capillary formation, and provides nutrition support for tumor metastasis, which is mainly caused by VEGF [280]. Hypoxia-induced VEGF in the tumor is the main stimulus in growing tumors [280].

While TGFβ targeting agents, such as galunisertib, have shown dramatic therapeutic effects in animal cancer models and in some cancer patients, it is still not clear how this therapeutic effect in cancer patients is achieved [279]. In this review, we describe the diverse targets of diabetes mellitus, that affect cancer cell biology. Small molecules that inhibit TGFβ-induced EMT are under development [282]. Silmitasertib is an inhibitor of protein kinase CK2, which is linked with TGFβ-induced EMT and is currently in clinical trials for bile duct cancer, and in preclinical development for hematological malignancies [283]. Another inhibitor, Galunisertib, is a strong TGFβ-type-I receptor kinase inhibitor, and reduces the size and growth of tumors [284]. The effect of EMT and its relationship to invasion and metastasis are highly context-specific and further studies are required to reveal its impact on cancer cell biology. EMT and EndMT inhibitors are valuable for clinical therapy with the greatest potential efficacy in treating cancers when used in conjunction with chemotherapeutic approaches.

DPP4 contributes to the disruption of glucose homeostasis and is involved in the development of hyperglycemia. However, in cancer, especially in the context of breast cancer, suppression of DPP4 has been shown to promote breast cancer progression in mice [135]. DPP4 inhibition promotes EMT, which is one of the key mechanisms that generates cancer-associated fibroblasts in cancer patients [135,137]. DPP4 inhibition-associated EMT affects neighboring cells by stimulating cellular transdifferentiation processes, the cumulative effects of which induce metastasis of several cancers. Despite this, several DPP4 inhibitors have shown diverse effects in organ protection.

In addition, SGLT2 expression is higher in diabetic conditions, which affects glucose homeostasis. Increased expression of SGLT2, which transports glucose from urine to kidney tubular epithelial cells, might be associated with disrupted glucose metabolism [161]. The disrupted central metabolism of diabetic tubules enhances myofibroblast formation through EMT and can involve the development of cancer in diabetic kidneys [160].

Similarly, COMT is an essential protein and protects from gestational diabetes, preeclampsia, and metabolic syndrome, and is involved in glucose homeostasis by affecting AMPK activation. Still, the role of AMPK in cancer biology is not clear. Cell-specific and tissue-specific roles of AMPK should be analyzed to provide a clearer picture of diabetes and cancer. In cancer patients, GC treatments are used to suppress inflammatory responses, however, they can also induce diabetes. Further studies are required to investigate the link between hyperglycemia and patient or cancer outcomes after GC treatment. It is recommended that all cancer patients receiving GC be screened for hyperglycemia with a random glucose test [260]. Either a search for non-steroidal GCs or alternative approaches could be used to test in cancer patients.

The majority of studies in diverse cell types suggest harmful effects of exogenous cortisol in diabetes and beneficial effects for cancer patients. However, the role of endogenous GC receptors in these diseases has not been explored. The protective effect of endothelial cell GR in vascular inflammation, atherosclerosis, sepsis, and organ fibrosis provides deeper insight into its organ-protective role, but more research is still needed in this area.

Future extension of these studies is the driving force behind the search for phytochemical and synthetic compounds such as flavonoids, chalcones, polyhydroquinolines, propiophenone derivatives, deoxyandrographolides, and thiazolidin-4-one derivatives. All of these have shown protective effects in mouse models of diabetes mellitus [285,286,287,288,289,290,291,292,293,294,295,296,297] and can be further tested and potentially utilized in the treatment of diverse forms of cancer cell progression and metastasis.
